# Mechanisms of B-Cell Oncogenesis Induced by Epstein-Barr Virus

**DOI:** 10.1128/JVI.00238-19

**Published:** 2019-06-14

**Authors:** Abhik Saha, Erle S. Robertson

**Affiliations:** a Department of Life Sciences, Presidency University, Kolkata, West Bengal, India; b Department of Otorhinolaryngology-Head and Neck Surgery, and the Tumor Virology Program, Abramson Comprehensive Cancer Center, Perelman School of Medicine at the University of Pennsylvania, Philadelphia, Pennsylvania, USA; University of Arizona

**Keywords:** B-cell lymphomas, Epstein-Barr virus, lymphoblastoid cell lines, tumor virology

## Abstract

Epstein-Barr virus (EBV) is a ubiquitous gammaherpesvirus which asymptomatically infects the majority of the world population. Under immunocompromised conditions, EBV can trigger human cancers of epithelial and lymphoid origin. The oncogenic potential of EBV is demonstrated by *in vitro* infection and transformation of quiescent B cells into lymphoblastoid cell lines (LCLs). These cell lines, along with primary infection using genetically engineered viral particles coupled with recent technological advancements, have elucidated the underlying mechanisms of EBV-induced B-cell lymphomagenesis.

## Abstract (precis)

Epstein-Barr virus (EBV) is a ubiquitous gammaherpesvirus which asymptomatically infects the majority of the world population. Under immunocompromised conditions, EBV can trigger human cancers of epithelial and lymphoid origin.

## INTRODUCTION

Epstein-Barr virus (EBV), also known as human herpesvirus 4 (HHV4), is highly immunogenic, with >95% of the world population found to be seropositive (1). Primary infection occurs in oropharyngeal epithelial cells; however, EBV predominantly infects B lymphocytes. Within the immunocompetent host, virus persists in naive memory B cells in a nonpathogenic state for the lifetime of the host. Intermittently, these virus-infected memory B cells differentiate into plasma cells ensuing in lytic-cycle activation, promoting infection of other resting B lymphocytes (2). In immunocompromised hosts, like postoperative organ transplant and HIV-infected patients, EBV infection demonstrated a strong association with several B-cell lymphomas (3). In addition, the list includes endemic/sporadic Burkitt's lymphoma (eBL/sBL), diffuse large B-cell lymphoma (DLBCL), classical Hodgkin’s lymphoma (cHL), primary central nervous system lymphoma (PCNSL), primary effusion lymphoma (PEL), and plasmablastic lymphoma (4, 5). These lymphomas exhibit a distinct expression pattern of latent genes. For example, EBV-associated posttransplant lymphoproliferative disorder (PTLD), PCNSL, and a fraction of DLBCL typically express a full repertoire of latent genes encoding six nuclear (EBNA1, -2, -3A, -3B, -3C, and -LP) and three membrane (LMP1, -2A, and -2B) proteins, along with several untranslated RNAs, recognized as the latency III program (6). Hodgkin’s lymphoma (HL) and Burkitt's lymphoma (BL) are characterized by a more restricted pattern of latent gene expression. While HL is associated with EBNA1, LMP1, and LMP2 expression (latency II), BL predominantly expresses EBNA1 (latency I) (7, 8). The coding and noncoding viral transcripts, with varied potencies, simultaneously affect multiple signaling cascades accompanied by genetic/epigenetic alterations leading to various EBV-driven B-cell lymphomas. The latency patterns of EBV gene expression in different B-cell lymphomas are summarized in Table 1.

**TABLE 1:** EBV-associated B-cell lymphomas and gene expression patterns

Lymphoma type^*a*^	Genes in latent expression	Latency program
Posttransplant B-lymphoproliferative disorder	EBNA1, EBNA2, EBNA3A, EBNA3B, EBNA3C, EBNALP, LMP1, LMP2A, LMP2B, EBER1, EBER2, and BHRF1 and BART miRNAs	III
HIV-linked B-lymphoproliferative disorder	EBNA1, EBNA2, EBNA3A, EBNA3B, EBNA3C, EBNALP, LMP1, LMP2A, LMP2B, EBER1, EBER2, and BHRF1 and BART miRNAs	III
Primary central nervous system lymphoma	EBNA1, EBNA2, EBNA3A, EBNA3B, EBNA3C, EBNALP, LMP1, LMP2A, LMP2B, EBER1, EBER2, and BHRF1 and BART miRNAs	III
eBL	EBNA1, EBER1, EBER2, and BART miRNAs	I
sBL	EBNA1, EBER1, EBER2, and BART miRNAs	I
HIV-linked Burkitt’s lymphoma	EBNA1, EBER1, EBER2, and BART miRNAs	I
cHL	EBNA1, LMP1, LMP2A, EBER1, EBER2, and BART miRNAs	II
HIV-linked Hodgkin’s lymphoma	EBNA1, LMP1, LMP2A, EBER1, EBER2, and BART miRNAs	II
DLBCL, NOS	EBNA1, LMP1, LMP2A, EBER1, EBER2, and BART miRNAs or all transcripts	II or III
DLBCL, PAL	EBNA1, EBNA2, EBNA3A, EBNA3B, EBNA3C, EBNALP, LMP1, LMP2A, LMP2B, EBER1, EBER2, and BHRF1 and BART miRNAs	III
DLBCL, HIV linked	EBNA1, EBER1, EBER2, and BART miRNAs or EBNA1, LMP1, LMP2A, EBER1, EBER2, and BART miRNAs or all transcripts	I, II, or III
PEL	EBNA1, EBER1, EBER2, and BART miRNAs	I
Plasmablastic lymphoma	EBNA1, EBER1, EBER2, and BART miRNAs	I

a eBL, endemic Burkitt’s lymphoma; sBL, sporadic Burkitt’s lymphoma; cHL, classical Hodgkin’s lymphoma (cHL); DLBCL, diffuse large B-cell lymphoma; NOS, not otherwise specified; PAL, pyothorax-associated lymphoma; PEL, primary effusion lymphoma.

Studies indicate that EBV also affects the lymphoma microenvironment, in which the latent oncoproteins manipulate cell machineries favoring the lymphoma cells for immune escape and proliferation (9, 10). The interaction between EBV-infected lymphoid cells and the tumor microenvironment offers promising therapeutic targets.

The transforming ability of EBV was discovered soon after its discovery from BL patient samples (11). The process of transformation of primary B cells *in vitro* has been used to establish EBV-transformed lymphoblastoid cell lines (LCLs) over many decades for genetic studies. These LCLs contain donor-specific genetic alterations. The viral gene expression pattern in LCLs is similar to that of B lymphoblasts isolated from patients having PTLDs, PCNSLs, and a fraction of DLBCLs (1, 6). Therefore, LCLs are being used as a surrogate *in vitro* model for studying the EBV-induced B-cell transformation process and subsequent lymphoma development. Since culturing cells under laboratory conditions for a long time may introduce further genomic instability, LCLs at early passage would be a better choice for functional validation and follow-up investigation into clinical samples.

## RECOMBINANT EBV BACmids

Using a bacterial artificial chromosome (BAC) system, the whole viral genome can be easily propagated in Escherichia coli (12, 13). Additionally, any desired mutations can be introduced into a specific viral gene locus. A number of labs across the globe utilized this strategy, delineating the precise function of a particular viral gene in B-cell transformation, or maintenance of outgrowth of transformed B-cell blasts. While, in most cases, the B95.8 EBV strain was utilized for the generation of BAC clones, there are examples where researchers used EBV-DNA from the BL line Akata (13). The EBV BAC clones, typically maintained in an epithelial cell background (HEK293T) under antibiotic selection, are induced by either overexpressing an immediate early viral protein, BZLF1 (14), or treating cells with chemical inducers: tetradecanoyl phorbol acetate (TPA), a protein kinase C inhibitor, along with sodium butyrate, a histone deacetylase (HDAC) inhibitor (15, 16). Occasionally, an immunosuppressive drug (FK506) is also used to facilitate the infection (17).

In our system, a green fluorescent protein (GFP) cassette was introduced to examine viral infection and to sort infected cells from uninfected populations (12). In other systems, several B-cell antigens are used to validate viral infection and the subsequent B-cell immortalization. These markers include surface antigen B-cell activation markers CD23, CD40, and CD44 and the intracellular B-cell proliferation marker *K_i_*-67 (12). CD40 plays an important role during B-cell activation by providing survival signals through its interaction with the CD40 ligand (CD154) expressed on the surface of activated T cells (18). Interestingly, LMP1 functionally mimics CD40 receptor-mediated signaling pathways and profoundly contributes to the formation of B-cell blasts (19). The early events of EBV infection in primary B lymphocytes provide a model for B-cell activation and downstream signaling processes, as well as the specific contributions of individual viral genes during B-cell transformation.

## B-CELL TRANSFORMATION

EBV-mediated B-cell transformation is associated with the global alteration of both viral and cell gene expression (20, 21). During the initial infection of primary B cells, almost all the genes, including lytic and latent ones, are expressed. While the DNA within the viral particle is unmethylated, in latently infected B cells, progressive methylation of the viral DNA regulates promoter usage and transcriptional repression (22). In the cell, viral DNA is associated with nucleosomes, collectively contributing to the restricted viral gene expression (23), while during the initial phase of infection, the entire viral DNA is accessible to the cellular transcription machinery; thus, many viral genes are simultaneously expressed (24). Importantly, during latent infection, EBV undergoes intermittent lytic replication, ensuring newer infection of the surrounding B cells. Additionally, lytic antigens are also closely associated with B-cell transformation (25), and accordingly, the removal of important lytic genes significantly affects B-cell transformation (23, 26–28).

Besides a differential viral gene expression pattern, cellular gene expression along with global epigenetic landscape are also largely affected (20, 21). For example, a drastic reduction in heterochromatin marks associated with transcriptional activation was observed during the initial phase of infection in quiescent B lymphocytes (20). In contrast, EBV infection leads to a global increase in promoter methylation of tumor suppressor genes (TSGs), leading to aberrant proliferation and transformation of the infected B cells (21).

## EBV TRANSFORMING ANTIGENS

**FIG 1:**
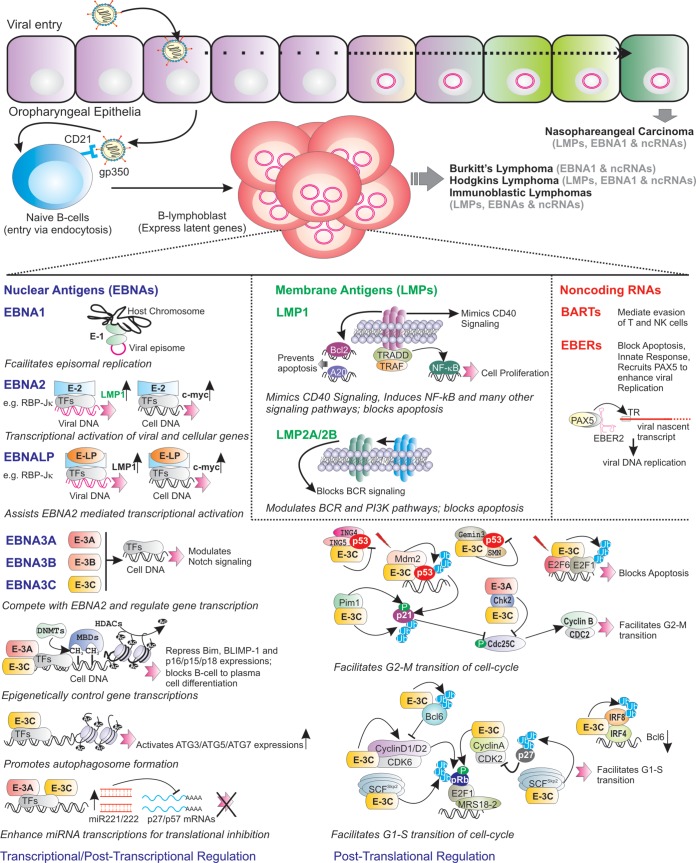
Salient features of EBV latent transcripts during B-cell transformation, followed by B-cell lymphoma development. After initial infection of oropharyngeal epithelial cells, EBV primarily infects the naive B lymphocytes. Subsequently, the infected B cells are growth transformed, expressing a subset of viral genes, with 6 nuclear antigens (EBNAs), 3 membrane proteins (LMPs), and several noncoding RNAs (EBERs and BARTs). EBNA1 binds to the episome origin of replication to allow viral genome replication. EBNA2 transcriptionally activates a number of viral (red) and cellular (black) genes through recruiting cell transcription factors (TFs), like RBP-Jκ, and induces cell growth. EBNALP promotes EBNA2-mediated gene transcription. EBNA3 proteins (EBNA3A, EBNA3B, and EBNA3C) modulate viral gene and Notch signaling by blocking EBNA2 association with RBP-Jκ. Both EBNA3A and EBNA3C recruit several epigenetic modifications (such as polycomb repressor complex 2 [PRC2]) to transcriptionally repress BIM, BLIMP-1, and p15, p16, and p18 expression and inhibit B-cell-to-plasma cell differentiation. Through epigenetic control, EBNA3C transactivates ATG3, ATG5, and ATG7 expression, thereby promoting autophagosome formation. EBNA3A and EBNA3C enhance miR221/222 transcription, which in turn block p27 and p57 translation. EBNA3C employs several mechanisms to block p53-mediated apoptotic activities. For example, EBNA3C recruits Mdm2 E3 ligase activity and stabilizes Gemin3 to enhance p53 degradation, and it competes with ING4 and ING5 binding to block p53-dependent apoptosis. EBNA3C enhances Pim-1-mediated p21 phosphorylation and degradation. Both EBNA3A and EBNA3C interact with Chk2 and facilitate the G_2_-M transition. In response to DNA damage signals, EBNA3C enhances E2F1 degradation, thereby blocking E2F1-mediated apoptosis. EBNA3C binds to E2F6 to block E2F1-mediated transcription. EBNA3C forms complexes and enhances the kinase activities of CyclinD1/CDK6, CyclinD2/CDK6, and CyclinA/CDK2 and augments pRb phosphorylation. EBNA3C recruits IRF4 to block Bcl6 expression and enhances IRF8 degradation. EBNA3C increases ubiquitin-proteasome-mediated degradation of hyperphosphorylated pRb, p27, and Bcl6, which facilitates the G_1_-S transition of the cell cycle. LMP1 mimics CD40 signaling and prevents apoptosis by upregulating bcl-2 and A20. LMP1, through interacting with tumor necrosis factor receptor (TNFR)-associated factors (TRAFs) and TNFR-associated death domain (TRADD) protein, constitutively induces NF-κB signaling pathway. LMP1 also activates JAK/STAT, ERK mitogen-activated protein kinase (MAPK), IRF, and Wnt signaling pathways. LMP2A blocks B-cell receptor (BCR) signaling, while LMP2B regulates LMP2A functions. EBV noncoding RNAs, the EBERs (EBER1 and EBER2), regulate the innate immune response and block apoptosis. EBER2 recruits PAX5 to the terminal repeat (TR) region of nascent viral transcript, which helps for viral lytic replication. BARTs mediate the evasion of T and NK cells during infection of B cells in peripheral blood lymphocytes.

Using various genetically engineered EBV and *in vitro* infection models, five viral latent antigens, EBNA2, EBNALP, EBNA3A, EBNA3C, and LMP1, are shown to be essential for efficient B-cell transformation (1, 6, 11). Other latent antigens and several noncoding RNAs also influence B-cell transformation and subsequent maintenance of B-cell outgrowth. Below, we discuss how modern genetic engineering strategies and *in vitro* infection, or transformed LCL model progressively revealed the importance of viral transcripts in B-cell transformation and subsequent development of B-cell lymphoma. Table 2 and Fig. 1 elucidate the major mechanisms associated with EBV latent transcripts.

**TABLE 2:** Impact of EBV latent antigens on B-cell transformation and subsequent lymphoma development

EBV latent protein	Function related to B-cell lymphomagenesis
EBNA1	Regulates viral DNA replication and transcription of a number of viral and cellular genes; facilitates p53 degradation and thereby promotes overall oncogenesis
EBNA2	One of the key viral transcription factors; in association with EBNALP, EBNA2 regulates transcription of several viral and cellular gene expression levels; essential for B-cell transformation
EBNALP	Transcriptional coactivator of EBNA2-mediated transcription of both viral and cellular genes; bypasses cell innate immune response; essential for B-cell transformation
EBNA3A	Along with EBNA3C, represses BIM and p14, p15, p16, and p18 gene transcription through epigenetic regulation; inhibits B-cell-to-plasma cell differentiation; essential for B-cell transformation
EBAN3B	Virus-encoded tumor suppressor protein
EBNA3C	Along with EBNA3A, represses BIM and p14, p15, p16, and p18 gene transcription through epigenetic regulation; facilitates G_1_-S and G_2_-M transitions of cell cycle; hijacks ubiquitin-proteasome pathway; inhibits p53-, E3F1-, and Bim-mediated apoptosis; activates autophagy; essential for B-cell transformation
LMP1	Functionally mimics CD40 signaling pathway; one of the major transcriptional regulators; constitutively activates NF-kB, JAK/STAT, ERK MAPK, IRF, and Wnt signaling pathways; stimulates bcl-2 and a20 expression to block apoptosis; essential for B-cell transformation
LMP2A	Functionally mimics BCR signaling pathway; blocks apoptosis; EBV latency regulation
LMP2B	Regulates LMP2A functions
EBERs	Most abundant noncoding viral RNAs present in all form of latency programs; affects innate immune response and gene expression; blocks PKR-dependent apoptosis
miRNAs	Transcribed from BART and BHRF1 loci; maintains latently infected B cells through blocking cellular apoptosis

### EBNA1.

Since EBNA1 is essential for DNA replication and maintenance of the viral latent genome, its expression expectedly has been demonstrated in all forms of latency programs (11). EBNA1 binding to a viral episomal origin of replication (OriP) recruits numerous cellular proteins, including DNA replication machinery ensuring appropriate duplication of the viral genome during each cell cycle. While in latency III, EBNA1 expression is maintained by the Cp promoter, in latency I, its expression is regulated by the Qp promoter (29). EBNA1 can coordinate the switch between different latency programs through promoter selection coupled with extensive epigenetic regulation (30). A genome-wide chromatin immunoprecipitation sequencing (ChIP-seq) analysis demonstrated that a chromosome insulator protein, CTCF, is involved in regulating the EBNA-mediated promoter switch and silencing of the Qp promoter in latency III-associated B cells (29).

Moreover, EBNA1 can induce the transcription of various cellular genes (31, 32) and contribute to the altered regulation of telomeres on cell chromosomes (33). The glycine-alanine repeat region of EBNA1 responsible for resistance to proteasome-mediated degradation plays an important role in the regulation of major histocompatibility complex class II (MHC-II) presentation to cytotoxic T lymphocytes (CTLs) (34, 35). This repetitive region also causes an indirect activation of c-Myc expression by a PI3 kinase (PI3K) signaling pathway (36). EBNA1 binding with ubiquitin-specific protease 7 (USP7) influences p53 and Mdm2 expression. This results in the regulation of antiapoptotic activity, possibly through promoting survivin expression levels (37, 38). Despite these critical activities, using recombinant virus, EBNA1 was shown to be not essential for *in vitro* B-cell transformation. However, EBNA1 expression enhanced the capability of the virus to drive B-cell transformation and the severity of associated lymphomas (39).

### EBNA2 and EBNALP.

EBNA2 and EBNALP are the first latent genes expressed after B-cell infection (6). EBNA2 represents the major viral transcription factor responsible for activating the expression of the entire repertoire of latent transcripts together with several host genes through employing cell transcription factors, RBP-Jκ and EBF1 (40). EBNALP simultaneously assists EBNA2-mediated transcriptional activity by blocking the NCoR and RBP-Jκ occupancy at the genome (41, 42). However, genome-wide ChIP-sequencing analyses in LCLs demonstrated that only one-third of the EBNALP sites are colocalized with EBNA2 sites, indicating the complicated nature of B-cell transformation induced by EBV infection (41, 42). EBNA2 most prominently contributes to the B-cell proliferation through transcriptional activation of approximately 300 cell genes, such as MYC and RUNX3 transcription (43, 44). Importantly, this transcriptional activation is regulated through superenhancers, characterized by dense clusters of several transcription factors coupled with enhanced signals for the H3K27ac histone activation mark (45). In contrast, EBNALP sites were occupied by RNA polymerase II, histone acetylase (HAT) p300, transcription factors such as SP1, PAX5, BATF, IRF4, PU.1, CTCF, RBP-J, and NF-κB, along with several histone activation marks, including H3K4me3, H3K27ac, H2Az, and H3K9ac (reference 46, and reviewed in reference 6).

### EBNA3 family proteins.

The EBNA3 family of proteins, consisting of EBNA3A, -3B, and -3C, represents transcription factors that precisely regulate host gene transcription and B-cell proliferation, particularly in an immunosuppressive setting (reviewed in references 47 and 48). It is believed that the EBNA3 gene family begun from cyclic duplications of an ancestral gene. Initial studies revealed that EBNA3A and EBNA3C, but not EBNA3B, cooperate with oncogenic Ha-Ras for transformation and immortalization of rat embryonic fibroblasts (49, 50). Later genetic studies revealed that EBNA3A and EBNA3C are necessary for B-cell transformation, whereas EBNA3B is dispensable (51, 52). An added complication to this idea came from a more recent finding that EBNA3B functions rather as a tumor suppressor in a humanized-mouse model NOD/SCID/γc^−/−^ through assisting T-cell surveillance (53). In fact, tumors induced by EBNA3B knockout virus demonstrated a lack of T-cell infiltrate and related activation of the chemokine CXCL10 (53). In contrast, EBNA3A and EBNA3C cooperatively act as predominant viral oncoproteins through regulating cellular gene transcription. Although functionally diverse, EBNA3 proteins share significant sequence similarity (∼30% at the N-terminal domain) and selection of cellular binding partners (48). Despite the sequence similarity, EBNA3C depletion can only be rescued by EBNA3C itself to maintain LCL outgrowth (54). This phenomenon is also true for EBNA3A (54). Initial experiments described that EBNA3 proteins negatively regulate EBNA2-mediated gene transcription through interaction with RBP-Jκ (55). Later, EBNA3A and EBNA3C were shown to interact with a long list of cellular proteins and transcription factors involved in regulating multiple cell signaling pathways. Additionally, although the functional relevance is still not clear in terms of B-cell lymphomagenesis, EBNA3C can form a complex with both EBNA3A and EBNA3B (56). The interacting partners for EBNA3C include transcription factors, chromatin modulators (both histone deacetylase and histone acetylase enzymes), cell cycle proteins involving G_1_-S and G_2_-M transitions, metastasis suppressor, posttranslational modifiers, E3-ubiquitin ligase, ubiquitin-specific proteases, unfolded protein response (UPR) regulator, cell tumor suppressors, and oncoproteins (references 57 and 58, and reviewed in references 47, 48, and 59). Similar to EBNA3C, EBNA3A also interacts with numerous cellular proteins, such as transcription insulators, cell cycle regulators, members of the ubiquitin protease complex, chaperones, and a number of proteins with unknown functions connecting to EBV-induced B-cell lymphomagenesis (reviewed in references 47 and 48). These viral proteins do not have specific binding sequence similarities, but regions associated with them are found to be occasionally functionally overlapping, indicating that both EBNA3A and EBNA3C employ complex oncogenic mechanisms with collaborative activities. Importantly, we and others using various genetically engineered BACmids expressing EBNA3C mutants, as well as transcomplementation assays, validated the *in vitro* biochemical studies and demonstrated the importance of these binding regions during initial infection or maintenance of LCL outgrowth (25, 49). For example, EBNA3C was shown to form a complex with Chk2 and thereby manipulates the G_2_/M phase of the cell cycle (60). Later, using a conditional knockout virus, EBNA3C was shown to block the ATM/Chk2-dependent DNA damage response during the initial phase of viral infection in B lymphocytes (61). Utilizing a similar strategy, both EBNA3A and EBNA3C were shown to concomitantly repress proapoptotic BIM (*BCL2L11*) and senescence-inducing p16^INK4A^ and p14^ARF^ (*CDKN2A*) by recruiting extensive epigenetic modifications (43, 54, 56, 62).

EBNA3A and EBNA3C block B-cell differentiation to a plasma cell phenotype through transcriptional activation of the cyclin-dependent kinase inhibitor p18^INK4c^ and the master transcriptional regulator of plasma cell differentiation BLIMP-1 (63). This helps establish a long-term latency and subsequent lymphoma development. Although EBNA3A and EBNA3C have similar oncogenic properties, genome-wide ChIP-sequencing analyses in LCLs revealed limited colocalization with a number of cellular transcription factors (62, 64). Most significantly, these two viral proteins regulate the transcription of many important cellular genes through recruitment of the IRF4-BATF complex (62, 64). In response to metabolic stress, EBNA3C, but not EBNA3A, activates autophagosome formation through transcriptional induction of several autophagy regulators, including ATG3, ATG5, and ATG7 (65). Moreover, similar to EBNA2 and EBNALP, EBNA3C among EBNA3 proteins acts as a potent regulator of viral gene transcription (66–68). EBNA3C-mediated coactivation of EBNA2 requires the PU.1 site, but not RBP-Jκ binding sites, in the LMP1 promoter (68). Overall, the EBNA3 proteins directly influence B-cell transformation and B-cell lymphoma development through targeting key cell signaling cascades, including the cell cycle, apoptosis, and autophagy. This involves direct protein-protein interaction, recruitment of chromatin remodeling factors (HATs, HDACs, and histone modification enzymes), translational control (microRNAs [miRNAs]), and the protein degradation machinery (chaperones, proteases, and ubiquitin ligases) (47, 48). Over the last decade, the employment of various technological developments, including genetically modified EBV either knocked out for each EBNA3 protein or conditionally expressed, and global transcriptomic and ChIP-seq analyses successfully demonstrated the importance of these proteins and offer potential therapeutic expansion against multiple B-cell lymphomas where EBNA3 proteins were expressed.

### Latent membrane proteins.

The transcripts of latent membrane proteins, LMP1, LMP2A, and LMP2B, are generated from a common viral locus with convergent and overlapping primary transcripts (69). LMP1 represents one of the major EBV-encoded oncoproteins mimicking the CD40 receptor signaling pathway (70). It is essential for EBV-induced B-cell transformation through the activation of multiple cellular pathways, such as the NF-κB, JNK, and p38 cascades (71–74). Using LCLs generated with either wild-type or CTCF binding domain knockout virus, it was demonstrated that CTCF plays an important role in regulating the transcription of LMPs from the OriP region and maintenance of episomal copy numbers during EBV latency (75). Unlike the nuclear antigens, LMPs particularly regulate the host immune response and thereby contribute to the activation and proliferation of the infected B cells, leading to B-cell lymphomas in the absence of immune surveillance (70). Using LMP1 knockout virus infection in a humanized mouse model, it has been clearly shown that activated T cells can substitute for the requirement of LMP1 expression in EBV-induced B-cell lymphomas by providing a source of CD40 signaling. However, compared to the LMP1 knockout virus, the wild-type virus can drive the formation of B-cell lymphomas more efficiently in this model (19, 70). LMP1 expression level varies in different EBV-associated B-cell lymphomas. For example, many EBV-induced AIDS-related lymphomas are associated with low LMP1 expression (19, 76), portrayed as a strategy for immune escape from activated CTLs, as LCLs with the highest level of LMP1 expression were demonstrated to enhance MHC I expression and subsequent killing by CTLs (70). Besides CD40 signaling, LMP1 also regulates cellular apoptosis through activation of the NF-κB pathway by elevating antiapoptotic Bcl2 expression (74, 77). Importantly, unlike the tumor necrosis factor receptor (TNFR), LMP1-mediated NF-κB activation is largely mediated via IRAK1 and TRAF6; IRAK1 is essential for both p38 activation and p65/RelA phosphorylation (78–80). LMP1 also modulates autophagy and the UPR network, affecting its own expression (76, 81, 82). Interestingly, LMP1-induced proapoptotic polycomb complex protein Bmi-1 is further recruited by EBNA3C for transcriptional repression of other genes (8, 83). Moreover, LMP1 expression is also controlled by EBNA3C in an EBNA2/RBP-Jκ-dependent manner (68).

LMP2B is a truncated isoform of LMP2A. While both LMP2A and LMP2B contain 12 transmembrane domains, LMP2B lacks the N-terminal cytoplasmic signaling domain (84). Although in B lymphocytes, LMP2A is tyrosine phosphorylated by the Src family kinase (such as Lyn and Syk), in epithelial cells, it is mediated by the C-terminal Src kinase, which is triggered by epithelial cell adhesion to extracellular matrix proteins (85). Through this domain, LMP2A acts as a functional homolog of the B-cell receptor (BCR), thereby promoting B-cell survival (86). The importance of this cytoplasmic domain was demonstrated by using an activation motif LMP2A mutant or the Syk inhibitor or Syk-specific small interfering RNA (7). LMP2A is absolutely necessary for growth transformation of germinal center-derived B cells, which are BCR negative (87). Unlike LMP1, LMP2A does not cause any adverse effect on B-cell maturation through the activation of immune surveillance (88). LMP2B negatively regulates LMP2A functions (89) and switches from latent to lytic activation through the depletion of LMP2A-mediated BCR cross-linking and restoration of Ca^2+^ mobilization (90). Interestingly, although none of these LMPs are essential to induce B-cell lymphomas in a humanized mouse model, the absence of LMPs caused a significant decline in the propensity of lymphoma development, indicating a plausible role in the initial phase of tumor growth (91). Interestingly, LMP2A can rescue LMP1-induced damage in the germinal center and promote cell cycle progression through accelerating c-Myc activity and p27^KIP1^ degradation (87, 88, 92).

### Noncoding viral transcripts.

In addition to nuclear and membrane-associated proteins, EBV expresses a variety of noncoding RNAs (ncRNAs) upon infecting B cells, namely, the EBV-encoded nonpolyadenylated RNAs (EBER1 and EBER2) and numerous miRNAs (reviewed in reference 93). Although most of these ncRNAs are not essential for B-cell transformation, they help with immune evasion and are abundantly expressed in the different types of latency programs, providing tools for viral detection in numerous EBV-associated malignancies. Overall, a somewhat contradictory role for EBERs in EBV-mediated B-cell transformation has been established (94). For example, the expression of EBERs increases colony formation, induces growth of B cells, and blocks PKR-dependent eukaryotic initiation factor 2 alpha (eIF2α) phosphorylation, resulting in a blockage of eIF2α-mediated inhibition of protein synthesis and resistance to alpha interferon (IFN-α)-induced apoptosis (95). EBERs also interact with several important cellular partners. For example, EBER1 interaction with ribosomal protein L22 regulates protein translation, EBER-mediated gene expression, and PKR-dependent apoptosis (96, 97). Interaction of EBERs with RIG-I, AU-rich element binding factor 1, and pattern recognition receptors activates the host innate immune responses (95, 98). In addition, EBER2 specifically recruits PAX5 to regulate LMP2A expression, which was also confirmed using an EBER2 mutant virus that showed lower LMP2A expression (99). Additional studies suggested that EBER1 and several viral miRNAs are exported from the infected cell in exosomes with functions related to activities in the surrounding cells (100).

Although EBV miRNAs are abundantly expressed in infected B lymphocytes, sometimes at a level as high as that of cell miRNAs, their precise role in B-cell transformation is not clear. Three BHRF1 and about 40 BART region miRNAs are expressed from different regions of the viral episome (reviewed in reference 93). While BART miRNAs are expressed in nearly all EBV-associated B-cell lymphomas, BHRF1-encoded miRNA expression is relatively restricted to different latency programs (101, 102). Expectedly, these viral miRNAs regulate the expression of a number of cellular genes. Although expendable, B cells infected with recombinant virus lacking viral miRNAs of the BHRF1 cluster resulted in a drastic reduction in their efficiency to support B-cell survival, proliferation, and transformation (103). Moreover, during the early phase of infection, viral miRNA expression levels are significantly higher than with transformed LCLs (104). In addition to their central role in immune evasion during the early phase of viral infection of the nascent B cells, many important cellular targets have been identified for BART and BHRF1 miRNAs, particularly those influencing apoptosis and B-cell proliferation (103). For example, while BHRF1 miRNAs are required for proficient B-cell transformation through targeting multiple tumor suppressor proteins, such as PTEN and p27^KIP1^, BART miRNAs block the expression of many tumor suppressor genes, including DICE1, PUMA, PTEN, and BCL2L11, to promote epithelial cell survival (105–108).

## FUTURE PERSPECTIVE

**FIG 2:**
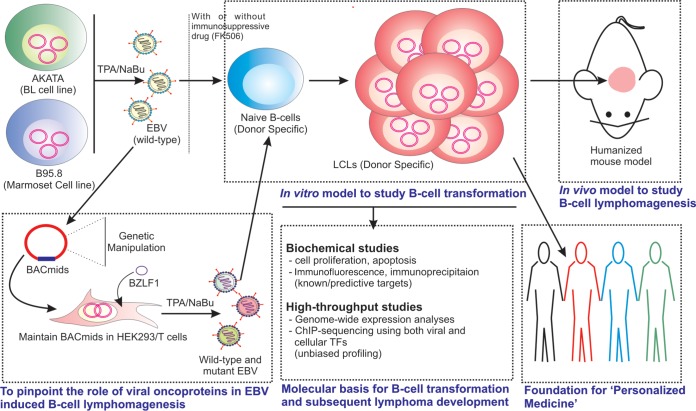
Systematic strategy for studying EBV-induced B-cell transformation and lymphomagenesis. Burkitt’s lymphoma (BL) cell line Akata or marmoset cell line B95.8 are used to generate virus particles and subsequent infection to nascent B lymphocytes in the absence or presence of an immunosuppressive drug, FK506. The addition of FK506 facilitates the transformation process though inhibiting T-cell-mediated immune surveillance. Alternatively, the whole-virus genome is cloned into BACmid and maintained in epithelial cells (HEK293 or HEK293T). In order to pinpoint the function of viral latent genes and respective domains, genetically engineered BACmids are used to transform naive B cells. B cells infected with wild-type virus are eventually growth transformed into continuously proliferating lymphoblastoid cell lines (LCLs), expressing latency III program with a full panel of viral latent transcripts, similar to many EBV-associated lymphomas in an HIV-infected population. Several biochemical assays and high-throughput strategies are employed to delineate the underlying mechanism of B-cell transformation and subsequent B-cell lymphoma development. Additionally, these LCLs are used to study EBV-induced B-cell lymphomagenesis in a humanized mouse model. Since the LCLs possess donor-specific genetic variations, they can provide an ideal *in vitro* model to study pharmacogenomics, leading to futuristic “personalized medicine.”

The ease of attaining EBV-transformed LCLs from practically any genetic background has led these cells to be used as a powerful tool for numerous investigations, as discussed above. Additionally, studies with LCLs have generated huge public resources on a genome-wide scale, highlighting critical regulation by multiple cell and viral transcription factors coupled with epigenetic alterations. Studies revealed a critical contribution of each viral oncoprotein and described the intricate nature of B-cell transformation and subsequent B-cell lymphoma development. Importantly, these LCLs are also being used as a preclinical model system for pharmacogenomic studies envisaging a drug response due to genetic predispositions along with epigenetic variations. Although LCLs are helpful for primary evaluation of a drug response and identification of biomarkers (reviewed in reference 109), experiments on human cancer cell line models, such as the NCI-60 panel (National Institutes of Health, USA) (110) and humanized mouse model (86) systems coupled with information from various omics data sets are also essential for subsequent validation prior to clinical trials. A number of LCL collections from diverse genetic backgrounds are now available for pharmacogenomics studies. Particularly, LCLs from the National Institute of General Medical Science (NIGMS) and National Human Genome Research Institute (NHGRI), including the LCLs used for the HapMap Project (111), have been extensively used. LCLs along with next-generation sequencing information from the ENCODE and the 1000 Genomes Project (112) have also been submitted into the NHGRI collection. Biobanking (113) is another strategy to maintain large LCL collections from population-based cohorts. In the coming years, LCLs would serve an important model system providing the foundation of “personalized medicine” (Fig. 2).

EBV was discovered more than 50 years ago and remains the most frequent persistent asymptomatic virus infection in humans suffering from several B-cell malignancies, particularly in an immunocompromised scenario. Nonetheless, great progress has been made in understanding the underlying oncogenic mechanisms by which EBV contributes to the development of different B-cell lymphomas. The comprehensive understanding of EBV biology gathered particularly in the last decade will certainly allow us to improve many aspects of clinical care regarding patients suffering from EBV-associated B-cell lymphomas. There are great opportunities to offer early diagnosis of different EBV-associated lymphomas with differentially expressed viral latent antigens, immunotherapy to specifically target EBV-infected B lymphocytes, and chemotherapy targeting potential cell pathways, as discussed above.
